# Lipodystrophy among HIV-infected patients: a cross-sectional study on impact on quality of life and mental health disorders

**DOI:** 10.1186/s12981-015-0061-z

**Published:** 2015-06-20

**Authors:** Charlotte M Verolet, Cécile Delhumeau-Cartier, Marlène Sartori, Simona Toma, Sophie Zawadynski, Minerva Becker, Enos Bernasconi, Laurence Toutous Trellu, Alexandra Calmy, The LIPO Group Metabolism

**Affiliations:** Department of Pediatrics, Children’s Hospital-University Hospitals of Geneva, 6 Rue Willy-Donzé, 1211 Geneva 14, Switzerland; HIV Unit, Department of Infectious Diseases, University Hospitals of Geneva, 4, Rue Gabrielle-Perret-Gentil, 1211 Geneva 4, Switzerland; Department of Dermatology and Venerology, University Hospitals of Geneva, 4, Rue Gabrielle-Perret-Gentil, 1211 Geneva 4, Switzerland; Department of Psychiatric Diseases, University Hospitals of Geneva, 4, Rue Gabrielle-Perret-Gentil, 1211 Geneva 4, Switzerland; Department of Nuclear Medicine, University Hospitals of Geneva, 4, Rue Gabrielle-Perret-Gentil, 1211 Geneva 4, Switzerland; Department of Radiology, University Hospitals of Geneva, 4, Rue Gabrielle-Perret-Gentil, 1211 Geneva 4, Switzerland; Department of Infectious Diseases, Hospital of Lugano, Via Tesserete 46, 6900 Lugano, Switzerland

**Keywords:** HIV infection, Lipodystrophy, Lipohypertrophy, Lipoatrophy, Quality of life, Mental health disorder, Depression, Anxiety

## Abstract

**Background:**

Lipodystrophy (LD) is a frequent adverse event of combination antiretroviral therapy (ART) and occurs mainly in patients exposed to first-generation antiretroviral drugs. The aim of this study was to explore and measure the interaction between LD, mental health, and quality of life of human immunodeficiency virus (HIV) positive individuals seen in a metabolic clinic.

**Methods:**

We conducted a single-site cross-sectional study including all HIV-infected patients attending the *LIPO group and metabolism* day clinic at the University Hospitals of Geneva, Switzerland between January 31, 2008 and November 28, 2013. Data on LD were prospectively collected using the HIV Outpatient Study (HOPS) score, the Lipodystrophy Case Definition (LDCD), ART regimens, anthropometric measures, imaging, and standardized questionnaires. Quality of life was evaluated using a visual analog scale of 0–100. Depression and anxiety were assessed using the Beck Depression Inventory and the State Trait Anxiety Inventory scales, respectively.

**Results:**

One hundred ninety-four patients (54.6% male; 45.4% female; median age, 50 years) on successful ART (median CD4 cell count, 569.0 cells/mm^3^; median viral load, 20 copies/mL) were evaluated. Among these, 62.7, 63.5 and 35.5% of patients reported at least one body site affected by fat hypertrophy, atrophy or both, respectively. Using the LDCD score conservative definition, including imaging and biological values, 57.8% were diagnosed with LD. Of these, 39.7% suffered from severe/very severe LD. Depression was reported by 35.6% of individuals; 51.9% had anxiety symptoms and 49.5% reported poor quality of life (defined as being inferior to 50% on a scale from 0 to 100%). LD (odds ratio (OR = 5.22, 95% confidence interval (CI) 1.07–25.37, p-value: 0.040), depression (OR = 4.67, 95% CI 1.08–20.31, p-value 0.040), and anxiety (OR = 7.83, 95% CI 1.91–32.03, p-value 0.004) all affected significantly the quality of life.

**Conclusions:**

LD, depression and anxiety were frequent features among HIV-infected individuals seen in the metabolic clinic and significantly impacted on their quality of life.

**Electronic supplementary material:**

The online version of this article (doi:10.1186/s12981-015-0061-z) contains supplementary material, which is available to authorized users.

## Background

Since the description of the first case of acquired immune deficiency syndrome (AIDS) in 1981, human immunodeficiency virus (HIV) infection has reached pandemic proportions and has continued to expand in worldwide; in 2012 an estimated of 35.3 million of peoples were infected by HIV [1]. Nucleoside reverse transcriptase inhibitors (NRTIs), followed by protease inhibitors (PIs), were the first antiretroviral (ARV) drugs used to treat HIV [2] and succeeded in decreasing mortality by an estimated of 4.2 million of deaths during the previous decade [1, 3, 4]. However, this therapeutic revolution was not without complications as multiple side-effects were associated with these first-generation ARV drugs. Among these, lipodystrophy (LD) was highlighted in 1998 by Carr et al. [5]. This syndrome comprises three phenotypes: lipohypertrophy (LH); lipoatrophy (LA); or a mixed pattern of both [5, 6]. LD can be associated also with metabolic disorders, therefore increasing the risk of cardiovascular disease [7].

There is currently no universal definition of LD. This implies significant variation in the prevalence rate, incidence, severity, and risk factors, which makes difficult any comparison between studies and across countries and ethnicities [7]. Diagnosis is based mainly on clinical observations by the patients themselves and by the physicians. To help meeting this challenge, the HIV Outpatients Study (HOPS) scale provides a subjective, but standardized LD evaluation using specific questions related to the clinical signs of the disorder [8]. Carr et al. developed a score in order to offer a more objective definition of LD, the Lipodystrophy Case Definition (LDCD), with a reported 79% sensitivity and 80% specificity [9, 10]. Nevertheless, this score does not allow differentiating between disease phenotypes [6].

Systematic reviews have discussed the impact of ARV agents and regimens on LD, but with discordant conclusions. If a clear relationship between NRTIs (thymidine analogs) and LA has been demonstrated, the causal role of specific drugs in the development of fat accumulation remains to be clarified [11, 12]. With the advent of newer drugs and the earlier detection of HIV, the prevalence of LD is now decreasing [11], although 48 weeks after the tests of a novel, potent NRTI, BMS-986001 a peripheral and central fat accumulation was observed [13]. However, the overall prevalence of LD remains high as many patients were exposed to first-generation molecules and damage caused to adipose tissue has only limited reversibility [14].

Several research groups have focused on the impact of LD on quality of life [15–23]. In addition, these reports have described discrepant results and conclusions regarding the interplay with mental health disorders, such as depression or anxiety [24–28].

This study aimed to identify the complex relationships between LD, mental health disorders, and the quality of life in patients attending the *LIPO group and metabolism* day clinic at the University Hospitals of Geneva, Switzerland.

## Methods

### Patients

All patients participating to the metabolic clinic (*LIPO group and metabolism*) at the University Hospitals of Geneva between January 31, 2008 and November 28, 2013 were included in the study. This multidisciplinary consultation (day hospital) is held once monthly and patients are referred to the clinic by their treating physician for several reasons, such as the evaluation of HIV-related or unrelated multiple medical co-morbidities, or LD management. A complete medical check-up is coordinated by the infectious diseases team and includes blood analyses, imaging [body composition and bone dual-energy X-ray absorptiometry (DXA), and thoracic vertebral level (T12) as well as lumbar vertebral level (L5) computed tomography (CT) single slice scans], and psychological, plastic surgery, dermatology, endocrinology, bone diseases and dietary consultations. Physicians from other medical specialties are available upon request e.g., nephrologist, endocrinologist, bone specialist, cardiologist, hepatologist. All patients provided written informed consent and the study protocol was approved by the clinical ethics committee of the University Hospitals of Geneva (CER #09017).

### Definitions

*LD* Patients and physicians both completed a questionnaire based on the HOPS scale [8]. Different parts of the patient body (arms, legs, cheeks and buttocks for signs of atrophy; abdomen, neck and breast for signs of hypertrophy) were evaluated and described as absent, subtle, moderate or severe. The different values were then stratified into five categories (none, all subtle, subtle/moderate, one severe, two or more severe). According to Lichtenstein et al. [8], this score was then classified in two strata; absent vs present and used like this in all the statistical analysis.

We used also the complete model of LDCD score, developed by Carr et al. and validated in a large cohort of patients [9], to determine the categories used to define the presence of LD [10]. LD was stratified in four classes, from very subtle/absent (<0 to 9.9 points) to severe/very severe (15 to >23 points). This score was then classified in two classes; absent (subtle/absent, subtle, moderate) versus present (severe/very severe) and used like this for all the statistical analysis. These definitions are presented in the Additional file 1: Table S1.

*Quality of life* Patients were asked by a trained psychologist to score their perceived quality of life on a 20 cm quantitative scale ranging from 0 to 100%. Zero corresponds to the “worst possible quality of life” and 100 to the “best possible quality of life”. This measure is often used in cognitive behavioral therapy and is a part of a wider score validated in different studies [29]. In our study, we used the short version of the score. Patients were classified as having a low (0–50%) or high quality of life (51–100%).

*Anxiety* was evaluated by a trained psychologist using the State Trait Anxiety Inventory (STAI Y-B) grading by a questionnaire with 20 items regarding the trait anxiety developed by Spielberger in 1983 [30]. A score equal or greater than 46 indicated the presence of anxiety symptoms.

*Depression* was measured by a trained psychologist using the Beck Depression Inventory (BDI) score [31], including a questionnaire with 21 items. Patients were considered to have depression when the score was above 19.

The waist was measured halfway between the last rib and the iliac crest, and the hips by the maximal buttock circumference.

### Imaging studies

A CT-scan consisting of two single slices of 6 mm thickness each obtained at the T12 and L5 level was performed in each patient in order to calculate the ratio between the visceral adipose tissue and the subcutaneous adipose tissue (VAT/SAT). Abdominal VAT and SAT were measures by contouring manually the areas with attenuation values of adipose tissue (range 190 Hounsfield Units to 30 Hounsfield Units) according to the literature [32]. Limb as well as leg fat measurement, both needed for LDCD score calculation, was obtained by Total Body Dual X Ray Absorbsometry (DXA) on a Discovery A Bone densitometer (Hologic Inc. Bedford, MA, USA). Total body scans were performed by DXA (Dual X Ray Absorbsometry) using the same Discovery A Bone densitometer all over the study period (Hologic Inc. Bedford, MA, USA). To assure reliability and stability of our device, Phantom was scanned daily; QC (quality control) data plot was verified online by a secured, centralized database program. ISCD (International Society of Clinical Densitometry) recommendations for precision assessment, scans acquisition and analysis are applied.

### Statistical analyses

We used a kappa test with an alpha threshold of 5% to measure the level of agreement between patients and physicians regarding the presence of fat loss or accumulation by using the HOPS score. A Chi-square test with an alpha threshold of 5% was used to verify if gender had an impact on qualitative variables (depression, anxiety, quality of life, and LDCD). We conducted a multivariate logistic regression model to identify risk factors for a low quality of life, including the severe LD (LDCD) variable and all other variables with a p value lower than 0.2 in the univariate model, to assess the specific contribution of severe LD (LDCD). To identify specific, potential confounding risk factors of quality of life, such as depression and anxiety, we developed an additional model including clinical risk factors considered to have a potential impact on quality of life in HIV population: age; gender; severe LD (LDCD), atrophy and hypertrophy observed by patients and physicians (HOPS); body mass index (≥30 kg/m^2^); HIV duration (≥10 years); United States Centers for Disease Control and Prevention HIV stage (B&C); CD4 count (≤300 cells/mm^3^); and HIV viral load (≥40 copy/mL). Finally, we performed the Hosmer–Lemeshow test, as well as the area under the ROC curve, to evaluate the overall fit of our two logistic regression models. Statistical analyses were carried out with STATA software, version 13.0 (StataCorp, College Station, TX, USA).

## Results

### Characteristics of the study population

One hundred ninety-four HIV+ individuals were included in the analysis median time since HIV diagnosis was 17 years and a median age of 50 years old [interquartile range (IQR) 44.0–55.0]; 88 (45.4%) were female and 108 (72.5%) were Caucasians (Table 1). Twenty patients (10.4%) were obese with a body mass index ≥30 kg/m^2^. Almost all patients (99.0%) were on antiretroviral therapy with a majority (88.5%) on nucleoside reverse transcriptase inhibitor (NRTI) treatment. Protease inhibitors (PIs) were used in 50.1% of the patients while 43.7% were taking non-nucleoside reverse transcriptase inhibitor (NNRTI). Among those, 36 (18.6%) were taking efavirenz (EFV), 34 (17.6%) on etravirine and 14 (7.2%) on nevirapine. Eighty-six percent had a viral load below the threshold of 40 copies/mL; median CD4 count was 569.0 cell/mm^3^ (IQR: 403.0–710.0). Sixty-eight patients (35.6%) reported signs of depression (moderate 23.6%; severe 12.0%). Of them, a psychiatric intervention was indicated in 89.0%, including a psychiatric evaluation in 22.9%, a psychopharmacologic treatment in 33.3%, and a psychotherapeutic treatment in 65.6%. Ninety-nine patients (51.9%) were anxious (moderate 27.8%; severe 24.1%). A psychiatric intervention was indicated in 83.9% of those, including a psychiatric evaluation in 18.3%, a psychopharmacologic treatment in 29.6%, and a psychotherapeutic treatment in 72.3%. However, we do not have the information on whether the patient came or not to his or her appointment. Finally, 93 (49.5%) mentioned poor quality of life (≤50%) (Table 1).

**Table 1:** Characteristics of the study population

	N missing	All patients	Quality of Life^a^		p-value
			Poor (≤50% visual score)	High (>50% visual score)	
		Median (IQR)	Median (IQR)	Median (IQR)	
		N = 194	N = 93	N = 95	
Patient characteristics					
Sex female, n (%)		88.0 (45.4)	50 (53.8)	34 (35.8)	0.013*
Age (years)		50.0 (44.0–55.0)	48.0 (43.0–52.0)	51.0 (45.0–59.0)	0.008*
Ethnicity	45				0.005*
Caucasians n (%)		108 (72.5)	42 (61.8)	62 (82.7)	
Africans n (%)		29 (19.5)	21 (30.9)	7 (9.3)	
Others n (%)		12 (8.0)	5 (7.3)	6 (8.0)	
Waist/hip ratio	9	0.9 (0.9–1.0)	0.9 (0.8–1.0)	0.9 (0.9–1.0)	0.105
VAT/SAT ratio^b,c^	9	0.5 (0.3–1.0)	0.4 (0.2–0.7)	0.5 (0.3–1.1)	0.009*
Body mass index (kg/m^2^)^d^	1	24.0 (22.0–27.0)	24.0 (22.0–27.0)	24.0 (22.0–26.0)	0.547
Triglycerides (mmol/l)		1.3 (0.9–2.2)	1.2 (0.8–1.9)	1.4 (1.0–2.4)	0.252
Total cholesterol (mmol/l)		5.1 (4.4–5.7)	5.0 (4.3–5.7)	5.1 (4.7–5.9)	0.105
HDL cholesterol (mmol/l)		1.2 (0.9–1.4)	1.1 (0.9–1.4)	1.2 (0.9–1.4)	0.795
HIV characteristics					
HIV duration (years)	20	17.0 (11.0–22.0)	15.5 (10.0–21.0)	18.5 (12.0–22.0)	0.099
CDC HIV stage^e^	14				0.362
A, n (%)		85.0 (47.2)	36.0 (41.9)	46.0 (52.3)	
B, n (%)		33.0 (18.3)	16.0 (18.6)	15.0 (17.0)	
C, n (%)		62.0 (34.4)	34.0 (39.5)	27.0 (30.7)	
CD4 (cells/mm^3^)	4	569.0 (403.0–710.0)	612.0 (411.5–752.0)	533.5 (394.0–700.0)	0.116
HIV viral load (copy/ml)	2	20.0 (20.0–40.0)	20.0 (20.0–40.0)	20 (20.0–40.0)	0.495
Antiretroviral therapy, n (%)		192.0 (99.0)	92.0 (98.9)	94.0 (98.9)	0.988
Protease inhibitors containing, n (%)	1	97.0 (50.5)	42.0 (46.1)	51.0 (54.3)	0.271
Integrase inhibitor containing, n (%)	1	41.0 (21.3)	17.0 (18.7)	24.0 (25.5)	0.262
NRTI^e^ containing, n (%)	1	170.0 (88.5)	83.0 (91.2)	81.0 (86.2)	0.280
NNRTI^f^ containing, n (%)	1	84.0 (43.7)	42.0 (46.1)	41.0 (43.6)	0.729
Efavirenz, n (%)	1	36.0 (18.6)	20.0 (22.0)	15.0 (16.0)	0.296
Etravirine, n (%)	1	34.0 (17.6)	16.0 (17.6)	18.0 (19.1)	0.783
Nevirapine, n (%)	1	14.0 (7.2)	6.0 (6.6)	8.0 (8.5)	0.622
Other regimens, n (%)	1	7.0 (3.6)	2.0 (2.1)	5.0 (5.5)	0.230
Questionnaire assessments					
Depression (BDI score)^g^	3				0.001*
Absent/very subtle (≤10 points), n (%)		61 (31.9)	11.0 (11.8)	50.0 (52.6)	
Subtle (11–18 points), n (%)		62 (32.5)	30.0 (32.3)	31.0 (32.6)	
Moderate (19–29 points), n (%)		45 (23.6)	31.0 (33.3)	12.0 (12.6)	
Severe/very severe (≥30 points), n (%)		23 (12.0)	21.0 (22.6)	2.0 (2.1)	
Anxiety (STAI-Y-B score)^g^	3				0.001*
Absent/very subtle (≤35 points), n (%)		35.0 (18.3)	3.0 (3.2)	32.0 (34.0)	
Subtle (36–45 points), n (%)		57.0 (29.8)	19.0 (20.4)	36.0 (38.3)	
Moderate (46–55 points), n (%)		53.0 (27.8)	34.0 (36.6)	19.0 (20.2)	
Severe/very severe (≥56 points), n (%)		46.0 (24.1)	31.0 (33.3)	6.0 (6.4)	
Lipodystrophy (LDCD score)^i^	59				
Absent/very subtle (≤9.9 points), n (%)		57.0 (42.2)	28.0 (46.7)	27.0 (38.6)	0.581
Subtle (10–14.9 points), n (%)		35.0 (25.9)	12.0 (20.0)	22.0 (31.4)	
Moderate (15–22.9 points), n (%)		12.0 (8.9)	6.0 (10.0)	5.0 (7.1)	
Severe/very severe (≥23 points), n (%)		31.0 (23.0)	14.0 (23.3)	16.0 (22.8)	

^a^Quality of Life Quantitative scale numbered 0%–100 according to the short version of EQ-5D-3L score.

^b^VAT: by CT-scan cross-section of 6 mm, level of L5: intra-abdominal adipose tissue.

^c^SAT: by CT-scan cross-section of 6 mm, level of L5: extra-abdominal adipose tissue.

^d^CDC HIV stage: according to CDC Classification System for HIV Infection by the United States Center for Disease Control and Prevention.

^e^NRTI: Nucleoside reverse transcriptase inhibitors.

^f^NNRTI: Non-nucleoside reverse transcriptase inhibitors.

^g^Depression: according to Beck Depression Inventory score by *Beck definition* (BDI).

^h^Anxiety: according to State Trait Anxiety Inventory by *Spielberger definition* (STAI-Y-B).

^i^LDCD: Lipodystrophy Case Definition by Carr et al.

* Significant *p*-value <0.05.

### Prevalence of LD

The prevalence of LD signs and symptoms were assessed using both the LDCD [10] and HOPS scores [8] (Additional file 1: Table S1). The LDCD score diagnosed LD among 57.8% individuals (from subtle to very severe LDCD score). Of these, 39.7% had imaging or biological features corresponding to severe/very severe LD (Table 1). According to the HOPS score, 62.7, 63.5 and 35.5% of patients subjectively reported at least one site affected by fat hypertrophy or atrophy or both, respectively. Patients and physicians were agree on 80.3% (substantial agreement, kappa 0.61; p-value 0.001) regarding the atrophy and on 79.1% (substantial agreement, kappa 0.57; p-value 0.001) regarding the hypertrophy (Table 2). The agreement regarding the atrophy between the two LD tests (HOPS score and LDCD score) was moderate (patients and physicians observations; 55.9 and 48.1% respectively, p-values <0.001 for both). Regarding hypertrophy, the results were not significant (patients and physicians observations (data not shown).

**Table 2:** Level of agreement between patients and physicians; HIV Outpatient Study (HOPS) score

	Patients observation	Physicians observation	Agreements (%)	Kappa	p-value	Interpretation
	N (%)	N (%)				
	N = 167	N = 144				
Atrophy	106 (63.5)	71 (49.3)	80.3	0.61	0.001*	Substantial
Hypertrophy	106 (62.7)	79 (54.9)	79.1	0.57	0.001*	Substantial

Atrophy/hypertrophy observed by patients/physicians: according to HOPS score by Lichtenchtein et al.

* Significant *p*-value <0.05.

### Mental health, quality of life, and LD stratified by gender

Women were more likely to present signs of depression or anxiety compared to men (46.0 versus 26.9%; p-value = 0.006; and 62.5 versus 42.7%; p-value = 0.006 for depression and anxiety, respectively) (Table 3). Although there was no gender difference in severe LD according to the LDCD score (p-value = 0.082). Men seem to be more affected by LA, while women suffered mainly from LH according to the HOPS score (Table 3). Moreover, women were more likely to score lower on quality of life than men (59.5 versus 41.4%, respectively; p-value = 0.013).

**Table 3:** Prevalence of mental health alterations and lipodystrophy symptoms by gender

	Male N (%)	Female N (%)	p value
Presence of depression (BDI score ≥19)^a^	28 (26.9)	40 (46.0)	0.006*
Presence of anxiety (STAI-Y-B score ≥46)^b^	44 (42.7)	55 (62.5)	0.006*
Poor QoL (≤50% visual score)^c^	43 (41.4)	50 (59.5)	0.013*
Severe LD (LDCD score ≥23 points)^d^	21 (28.8)	10 (16.1)	0.082
HOPS score:^e^			
Atrophy observed by patients	66 (72.5)	40 (52.6)	0.008*
Atrophy observed by physicians	46 (60.0)	25 (37.3)	0.007*
Hypertrophy observed by patients	47 (51.1)	59 (76.6)	0.001*
Hypertrophy observed by physicians	35 (46.7)	44 (63.8)	0.039*

^a^Presence of depression: according to Beck Depression Inventory score by *Beck definition* (BDI).

^b^Presence of anxiety: according to State Trait Anxiety Inventory by *Spielberger definition* (STAI-Y-B).

^c^Quality of Life Quantitative scale numbered 0%–100 according to the short version of EQ-5D-3L score.

^d^Severe LD: according to LDCD score Lipodystrophy Case Definition by *Carr* et al.

^e^Atrophy/Hypertrophy observed by patients/physicians: according to HOPS score by *Lichtenchtein* et al.

* Significant *p*-value <0.05.

### Factors associated with poor quality of life (QoL)

Among the 188 patients who completed the questionnaire, 93 (49.5%) described a poor quality of life (median (IQR): 40.0 (30.0–50.0) with a visual score of <50% [29]. Although none of the demographic variables was found to be associated with poor quality of life in the multivariate models (M1, M2, Table 4), HIV-related variables, such as duration of HIV diagnosis (categorized by ≥10 years) and the presence of a detectable viral load (categorized by ≥40 copy/mL) both were associated with a poor quality of life; the shorter the time after HIV diagnosis, the worse the quality life (Table 4). Fat atrophy observed by patients and physicians was associated with a poor quality of life in the univariate model, but was no longer statistically significant in the adjusted analyses. Symptoms of depression [M1: OR = 3.62 (95% CI 0.99–13.27); M2: OR = 4.67 (95% CI 1.08–20.31), anxiety features (M1: OR = 7.10 (95% CI 1.99–25.30); M2: OR = 7.83 (95% CI 1.91–32.03)), and severe LD (LDCD) (M1: OR = 6.25 (95% CI:1.33–29.40); M2: OR = 5.22 (95% CI: 1.07–25.37)] were all associated with a poor quality of life in the univariate model (Table 4). They remained statistically significant in both multivariate analyses, apart from depression, which was significantly associated in the second model only (Table 4). No interaction between mental health disorders (depression or anxiety) and LD has been highlighted within the multivariate logistic regression model M2 (data not shown). Finally, we have assumed that the two models are correctly specified with no statistical difference and are in concordance (for M1 and M2, respectively: Hosmer–Lemeshow test: p-value: 0.521 and 0.286; area under the ROC curve 0.847 and 0.863; p-value: 0.338).

**Table 4:** Risk factors for poor quality of life

	Poor quality of life^a^ (≤50% visual score)					
	Univariate regression		M1: p-value ≤0.2 in univariate model regression		M2: Clinical risk factors	
	OR (95%IC)	p-values	OR (95%IC)	p-values	OR (95%IC)	p-values
Patient characteristics						
Age (≥50 years)	0.62 (0.35–1.11)	0.109*	1.16 (0.38–3.54)	0.794	1.73 (0.52–5.68)	0.365
Gender (F)	2.09 (1.16–3.74)	0.014*	1.05 (0.35–3.10)	0.930	1.49 (0.46–4.78)	0.502
BMI (≥30 kg/m^2^)^b^	0.90 (0.53–2.33)	0.828	–	–	1.01 (0.14–7.18)	0.988
Triglycerides (≥2.0 mmol/L)	0.62 (0.33–1.16)	0.135*	0.60 (0.19–1.94)	0.397		
HIV characteristics						
HIV duration (≥ 10 years)	0.43 (0.21–0.92)	0.030*	0.14 (0.03–0.79)	0.026**	0.10 (0.01–0.63)	0.014**
CDC HIV stage (B&C)^c^	1.52 (0.84-–2.77)	0.170*	0.81 (0.27–2.39)	0.697	0.95 (0.30–3.00)	0.936
CD4 (≤300 cells/mm^3^)	0.57 (0.20–1.64)	0.300	–	–	0.09 (0.01–1.04)	0.054
HIV viral load (≥40 copy/mL)	2.55 (1.04–6.2)	0.039*	10.4 (1.73–2.55)	0.011**	10.39 (1.79–60.16)	0.009**
Current use of efavirenz	1.46 (0.69–3.06)	0.316	–	–		
Questionnaire assessments						
Severe LD (LDCD score ≥23 points)^d^	1.02 (0.45–2.33)	0.949	6.25 (1.33–29.40)	0.020**	5.22 (1.07–25.37)	0.040**
HOPS score:^e^						
Atrophy observed by patients	0.51 (0.27–0.99)	0.047*	0.49 (0.12–2.07)	0.334	0.27 (0.05–1.40)	0.120
Atrophy observed by physicians	0.51 (0.26–1.00)	0.051*	1.13 (0.30–4.26)	0.860	1.47 (0.37–5.84)	0.580
Hypertrophy observed by patients	1.37 (0.73–2.58)	0.325	–	–	0.50 (0.10–2.49)	0.396
Hypertrophy observed by physicians	0.75 (0.38–1.47)	0.400	–	–	0.56 (0.13–2.39)	0.438
Presence of depression (BDI score ≥19)^f^	7.33 (3.64–14.77)	0.001*	3.62 (0.99–13.27)	0.053	4.67 (1.08–20.31)	0.040**
Presence of anxiety (STAI-Y-B score ≥46)^g^	8.44 (4.37–16.30)	0.001*	7.10 (1.99–25.30)	0.003**	7.83 (1.91–32.03)	0.004**

^a^Quality of Life Quantitative scale numbered 0%–100 according to the short version of EQ-5D-3L score.

^b^BMI: body mass index (bodyweight in kilogram/(height in meter).

^c^CDC HIV stage: according to CDC Classification System for HIV Infection used by the United States Center for Disease Control and Prevention.

^d^Severe LD: according to LDCD score Lipodystrophy Case Definition by Carr et al.

^e^Atrophy/Hypertrophy observed by patients/physicians: according to HOPS score by Lichtenchtein et al.

^f^Presence of depression: according to Beck Depression Inventory score by *Beck definition* (BDI).

^g^Presence of anxiety: according to State Trait Anxiety Inventory by *Spielberger definition* (STAI-Y-B).

* Significant *p*-value in univariate model <0.2.

** Significant *p*-value <0.05.

## Discussion

Results of our study indicate a high frequency of LD and mental health disorders among HIV-infected men and women on successful ART presenting at the *LIPO group and metabolism* day clinic. Almost half of all patients considered themselves as suffering from a poor quality of life. Severe LD, depression, and anxiety were all associated with poor quality of life. This high level of LD frequency is partly due because the patients are referred to the day clinic by their treating physician.

Through these findings, we demonstrate that the objective signs of LD, based on a validated score (LDCD) and including morphologic data and metabolic values, are associated with a decreased quality of life and with the presence of mental health disorders. Fat loss observed by the patient and the physician (HOPS score) was also associated with a poor quality of life, but only in the univariate analysis.

Our results are consistent with several previous studies. First, some authors have highlighted a poor quality of life, severe stigma, and lower values in social and psychological scores regarding HIV-infected patients with or without reported LD [33, 34]. Of note, treatment of mental health disorders improved quality of life [33]. Secondly, other studies emphasized an association between LD and a negative effect on body image [18, 28, 34]. Indeed, because of body changes, stigma remains, leading to poor self esteem [20]. This leads to social isolation [24], decrease of sexual activity [26], and mental health disorders, such as anxiety and depression [35], which contribute to poor quality of life [21]. Guaraldi et al. evaluated LD by three scores, i.e., the Multicenter AIDS Cohort Study score (MACS)-HOPS-LDCD. The results of their study is consistent with our finding that LD is related to a poor quality of life [19]. Similar to other reports, they observed that the presence of LD can lead to ART discontinuation [19, 36, 37]. By contrast, a large study showed that individuals suffering from morphological alterations, such as LD, maintain a good adherence regarding ART [38].

Other studies show discordant results. Quality of life scales and psychological questionnaire often change from one study to another (i.e.: Health Related QoL (HRQoL) [13, 16, 18]; Profil der Lebensqualität Chronischkranker (PLC) [19], HIVspecific QoL [32]) and thus complicates comparison between the targeted population. In addition, study objectives vary (i.e.: cosmetic result, antiretroviral tolerance, global wellbeing, social isolation) and may influence the results. Barata et al. assessed a psychological questionnaire in outpatients with and without LD and found no difference in mental health disorders between the two groups [25]. Rajagopalan et al. found similar results regarding quality of life; however, a subgroup analysis showed that patients with LD, particularly homosexual men and patients undergoing psychiatric treatment, suffered from a low QoL [22].

LD remains a prevalent adverse event, despite the advent of new generation ARV drugs [18]. A recent French observational multicenter study showed that more than half of all patients on long-term effective ART suffer from disturbing and stigmatizing facial lipoatrophy. Congruent with reports from other groups, its prevalence is around 30% in patients who recently started their ARV treatment (1–5 years) [39]; the extent to which signs of LH reflect the physiological aging process is debated [40]. In our cohort, no difference was observed in the prevalence of LA and LH among individuals aged 50 or more and younger patients.

In a recent analysis of 15,275 participants to the Swiss HIV Cohort Study, Keiser et al. describe the suicide rate in HIV patients from 1998 to 2008. Despite a significant decrease rate of suicide between the pre era ARV drugs and the late era, suicides are more frequent in HIV population compared to general population [41]. In 62% of them, mental health disorders, as depression and anxiety, have been highlighted after the HIV diagnosis [41]. HIV infection and its possible impact on mental health can be compared to other chronic diseases, such as diabetes, asthma, or chronic kidney disease [42–47]. Indeed, a similar relationship has been highlighted in a recent study showing a major increase of anxiety symptoms as well as depression in patients with chronic obstructive pulmonary syndrome disease [48]. Interestingly, although the symptoms related to pulmonary disease could not be improved, recognition of psychological distress by the physician led to an enhancement of quality of life [48].

This study shows a complex interplay between quality of life, mental health disorders, and signs of LD. Severe LD and mental health disorders were all associated with a subjective impairment of quality of life. Moreover, individuals with a more recent diagnosis (<10 years) or those with a detectable viral load (≥40 copy/mL), more likely due to insufficient adherence to treatment, had also a poor quality of life. Therefore, psychological issues in HIV chronic infection is of major importance for daily constraints associated with the disease, adherence to therapy, and quality of life.

Women were more likely to express signs of depression and anxiety as found in other studies [49, 50]. Depressive symptoms are more than two-fold than those reported in the general population, while the number of men and women patients with anxiety symptoms is multiplied by 2.5 [51]. Men were more likely addressed to our group for LA and women for LH. We may speculate that the acceptability of LA and LH phenotype is gender-influenced.

Our study has some limitations. First, we used a modified quality of life score. This scale registers the self-estimation of the patient within a very specific timeframe. For this reason, a various number of independent variables can interfere with this estimate. We arbitrarily defined poor quality of life as between 0–50 and good quality of life between 51–100 and these thresholds could be challenged. However, this measure is simple to evaluate, and easily repeatable. Moreover, the QoL score that we used in our study has the advantage to be used directly during the clinical interview and measure synthetically the psychic pain in a biopsychosocial situation. As the validated Visual Analog Scale (VAS) largely used for pain appreciation, our QoL scale measure a subjective definition. Secondly, LD assessment remains difficult because of the lack of a gold standard definition. However, we consider that the use of two different scores, one based on the physician and patient perceptions and the other based on objective features, allowed us to capture with sufficient precision the prevalence and the severity of LD in this population. We were able to assess LD with great precision, using multiple and routinely-performed tools during the patient one-day visits to the day hospital. These included 191 of validated questionnaires, representing 98.5% of the patients included in the cohort, and 135 imaging (69.6%) (CT and DXA scans), as well as biological values, which are all features related to the LD. Regarding the HOPS score, there was good concordance between physician and patient assessments for fat atrophy and hypertrophy. Moreover, although the study population represented selected individuals referred by their practicing physician, it included both men and women of diverse ethnicity, thus making our results more consistent.

## Conclusions

The majority of the HIV-infected patients seen in the metabolic clinic in Geneva describe, at least, one body site affected by LA or LH and are diagnosed with LD regarding the LDCD score. Depression as well as anxiety feature are also very frequent in our studied population, and, with the presence of LD, are related to a poor quality of life, leading to psycho-social consequences. Mental health and a simple score for quality of life should be routinely assessed among HIV-infected individuals, including those on effective ART, in order to recognize early signs and symptoms of psychological distress and to help patients have better outcomes in their daily life.

## Additional file

Additional file 1:
**Table S1.** Definition of lipodystrophy used in the LIPO group and metabolism day hospital.

## Abbreviations

AIDS: acquired immune deficiency syndrome
HIV: human immunodeficiency virus
NRTIs: nucleoside reverse transcriptase inhibitors
NNRTIs: non-nucleoside reverse transcriptase inhibitors
PIs: protease inhibitors
ARV: antiretroviral
ART: antiretroviral therapy
LD: lipodystrophy
LH: lipohypertrophy
LA: lipoatrophy
HOPS: HIV outpatients study
LDCD: lipodystrophy case definition
QoL: quality of life
CDC HIV stage: CDC Classification System for HIV Infection used by the United States Center for Disease Control and Prevention
DXA: bone dual-energy X-ray absorptiometry
CT: computed tomography
STAI Y-B: state trait anxiety inventory
BDI: beck depression inventory
VAT: visceral adipose tissue
SAT: subcutaneous adipose tissue
CI: confidence interval
OR: odds ratio
